# Supplementary material for: Investigations on Anticancer Potentials by DNA Binding and Cytotoxicity Studies for Newly Synthesized and Characterized Imidazolidine and Thiazolidine-Based Isatin Derivatives
Source: Molecules. 2022 Jan 6;27(2):354. doi: 10.3390/molecules27020354 (PMC8778244; doi:10.3390/molecules27020354)
Supplement: Supplementary file 1 [file molecules-27-00354-s001.zip › molecules-1513005-supplementary.pdf]

Supplementary material

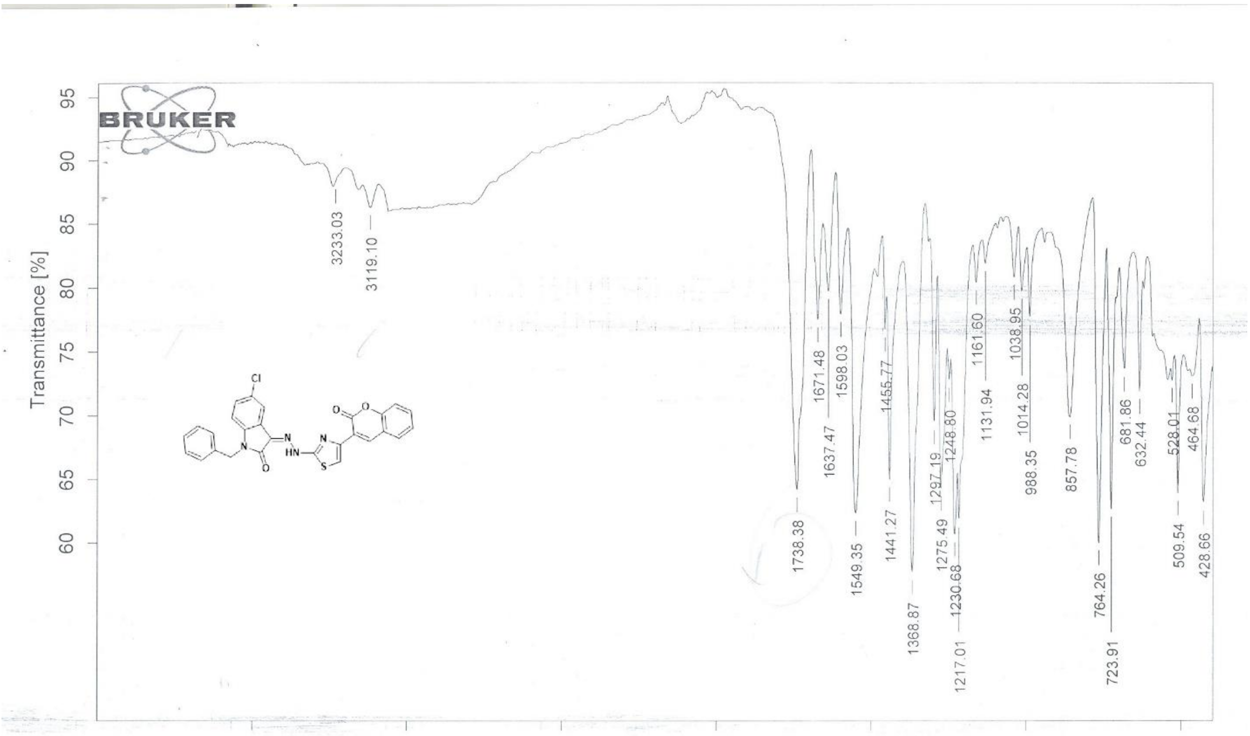

Figure S1. FT-IR spectrum of IST-01.

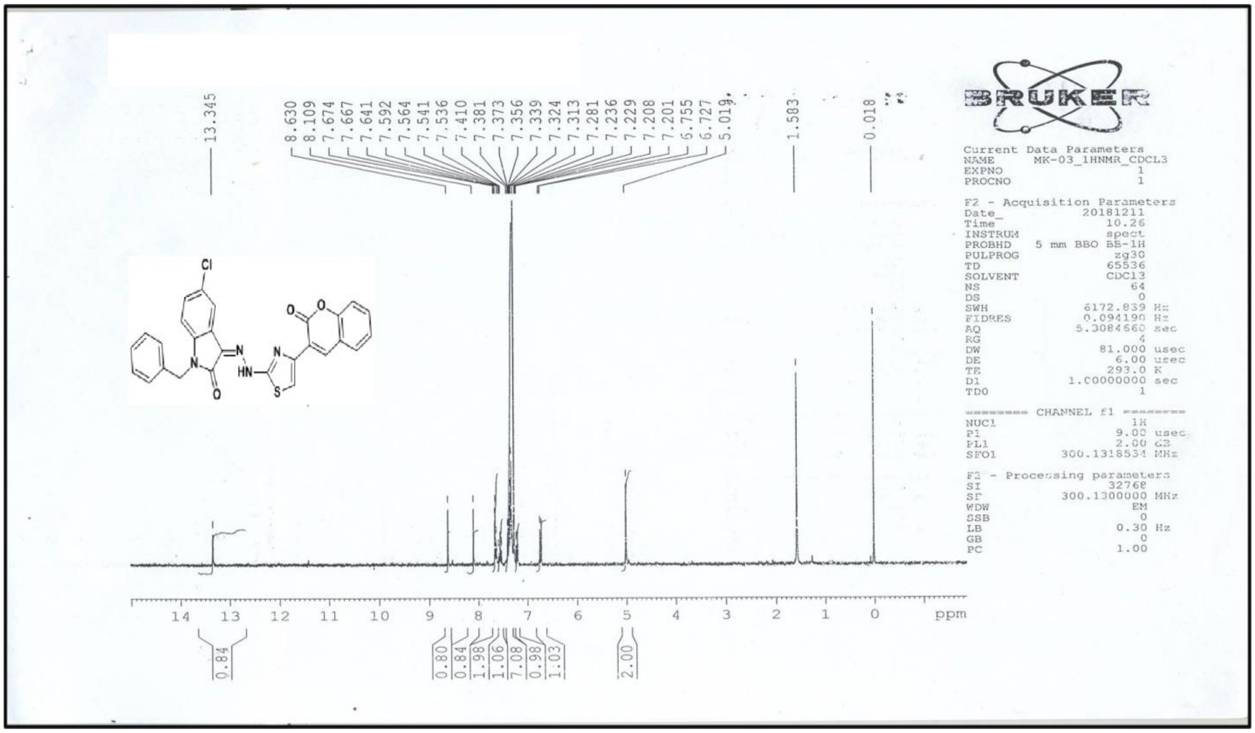

Figure S2. ^{1}H-NMR spectrum of IST-01.

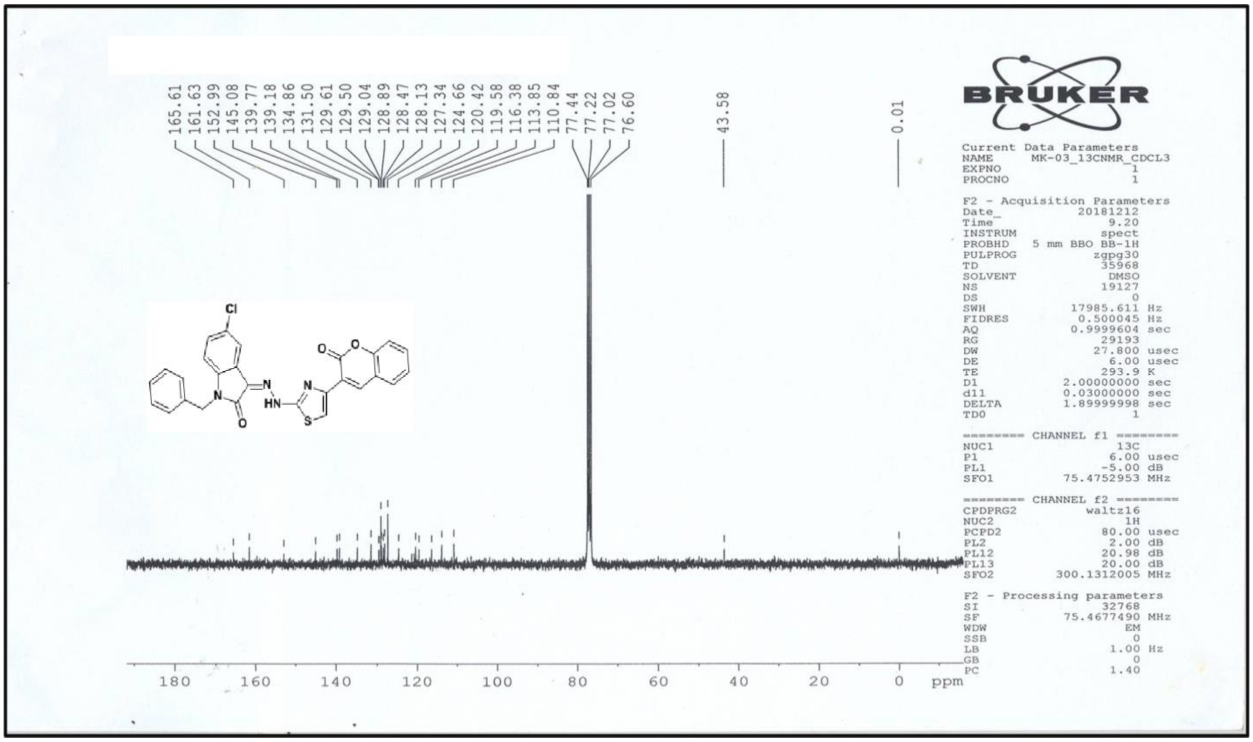

Figure S3. ^{13}C-NMR spectrum of IST-01.

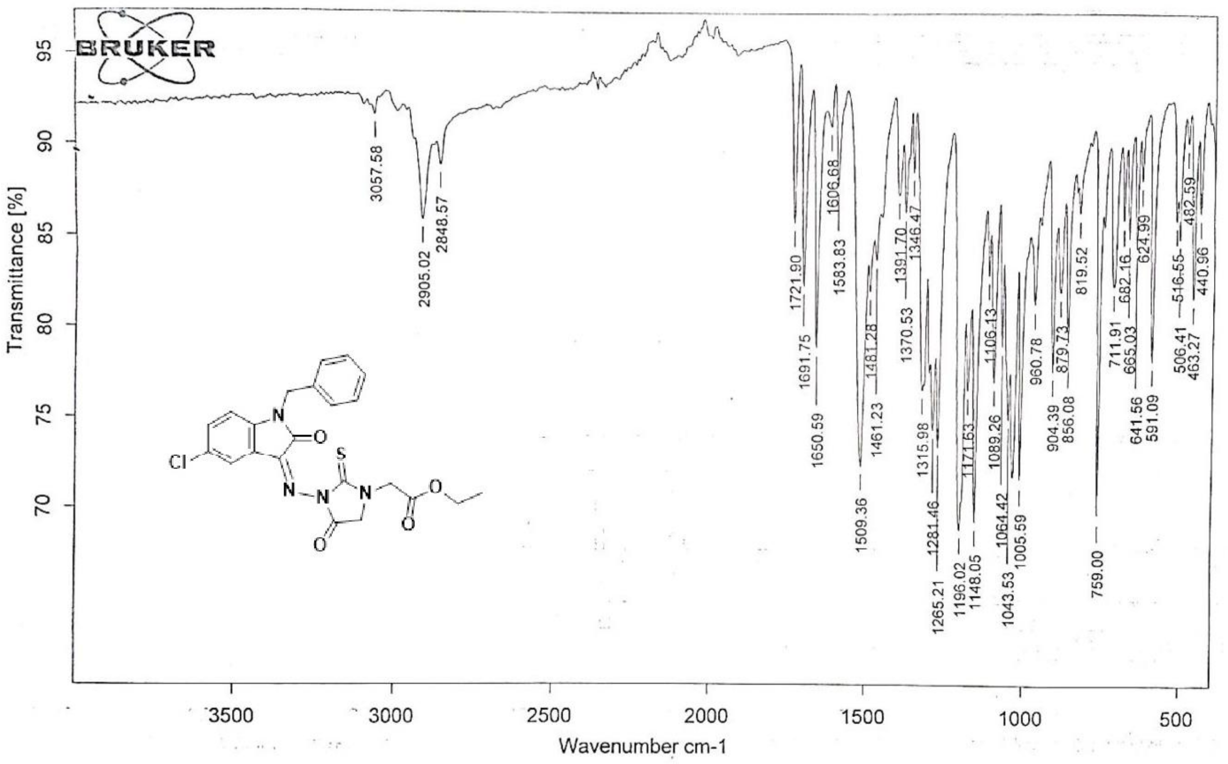

Figure S4. FT-IR spectrum of IST-02.

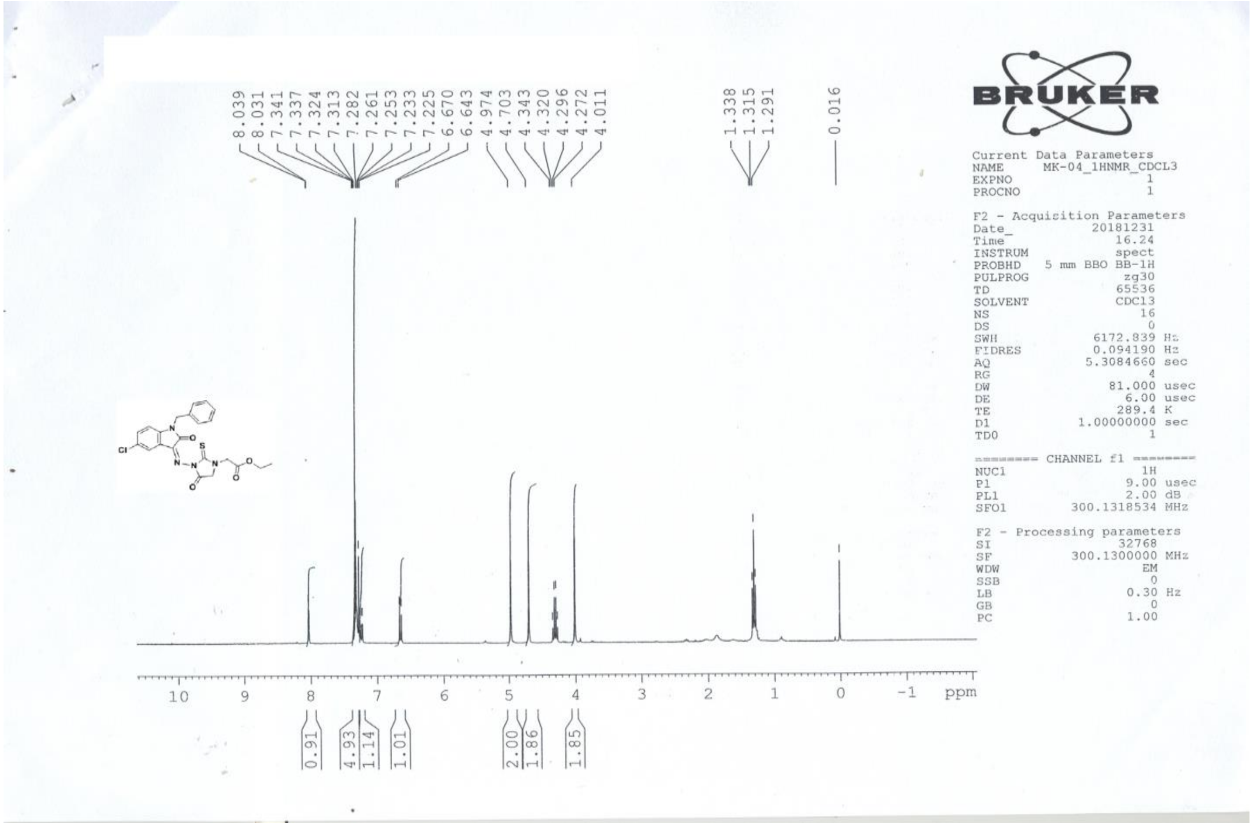

Figure S5. ^{1}H-NMR spectrum of IST-02.

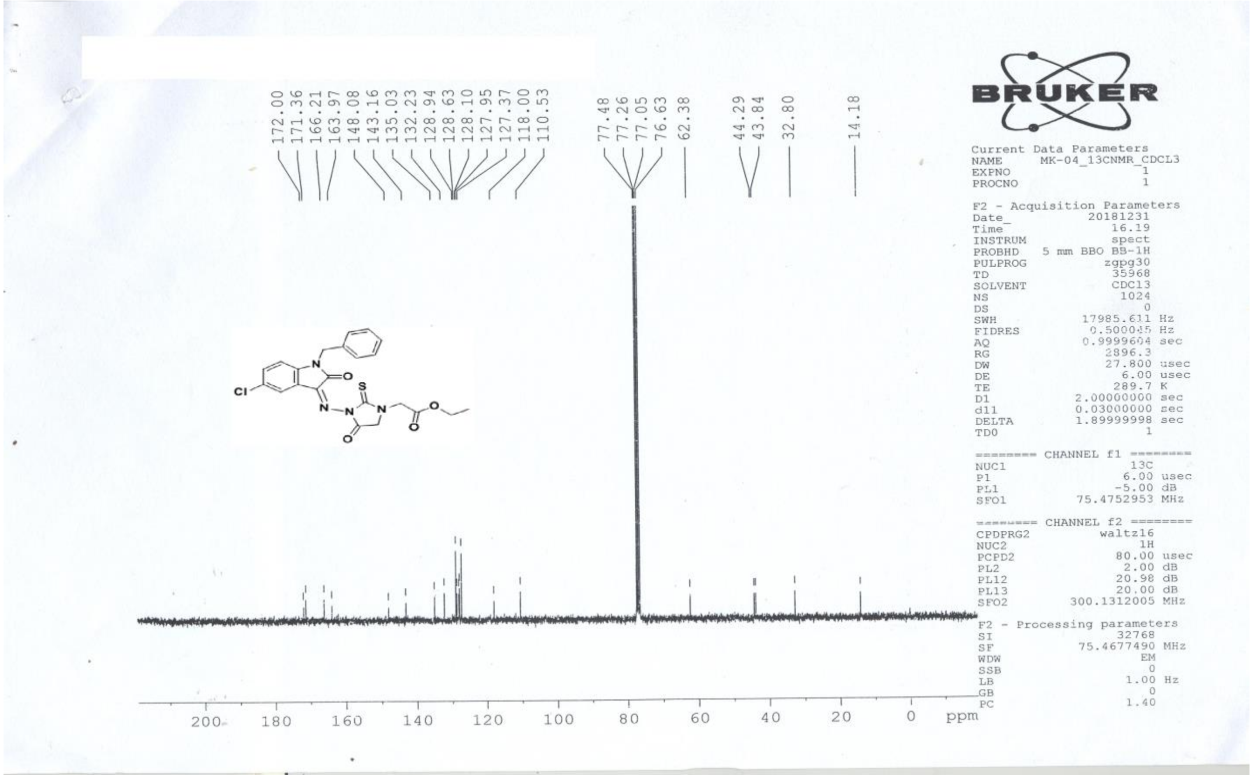

Figure S6. ^{13}C-NMR spectrum of IST-02.

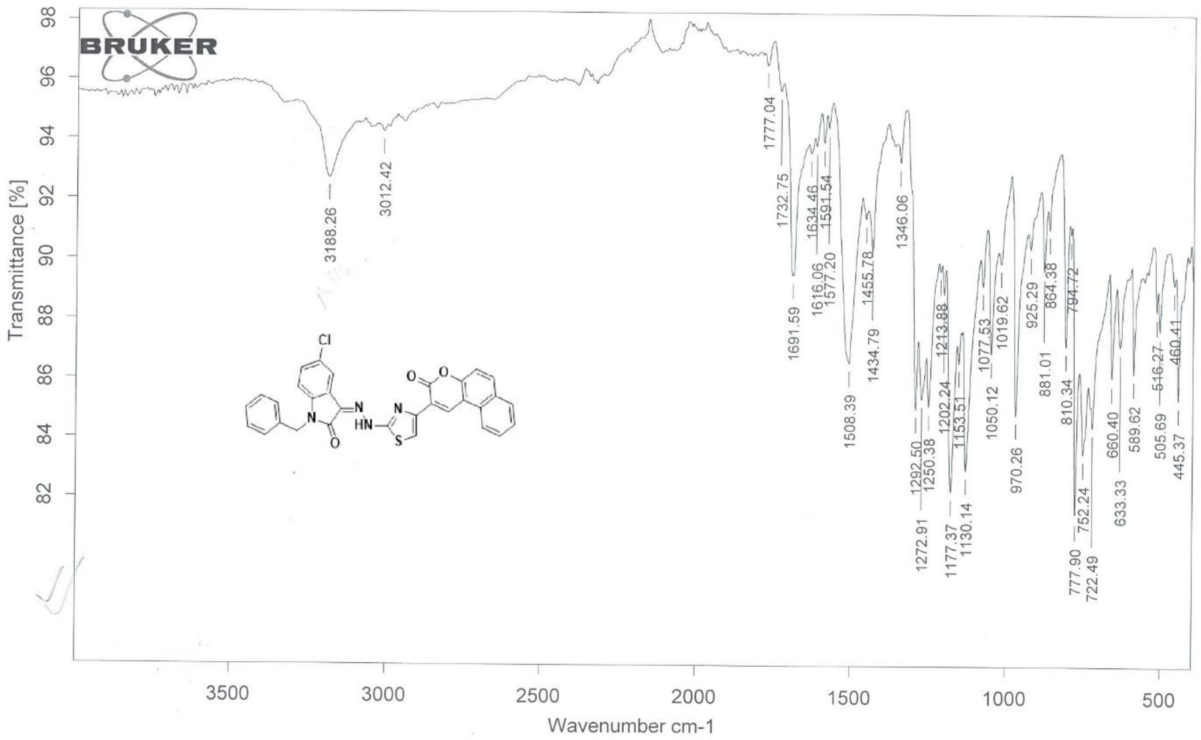

Figure S7. FT-IR spectrum of IST-03.

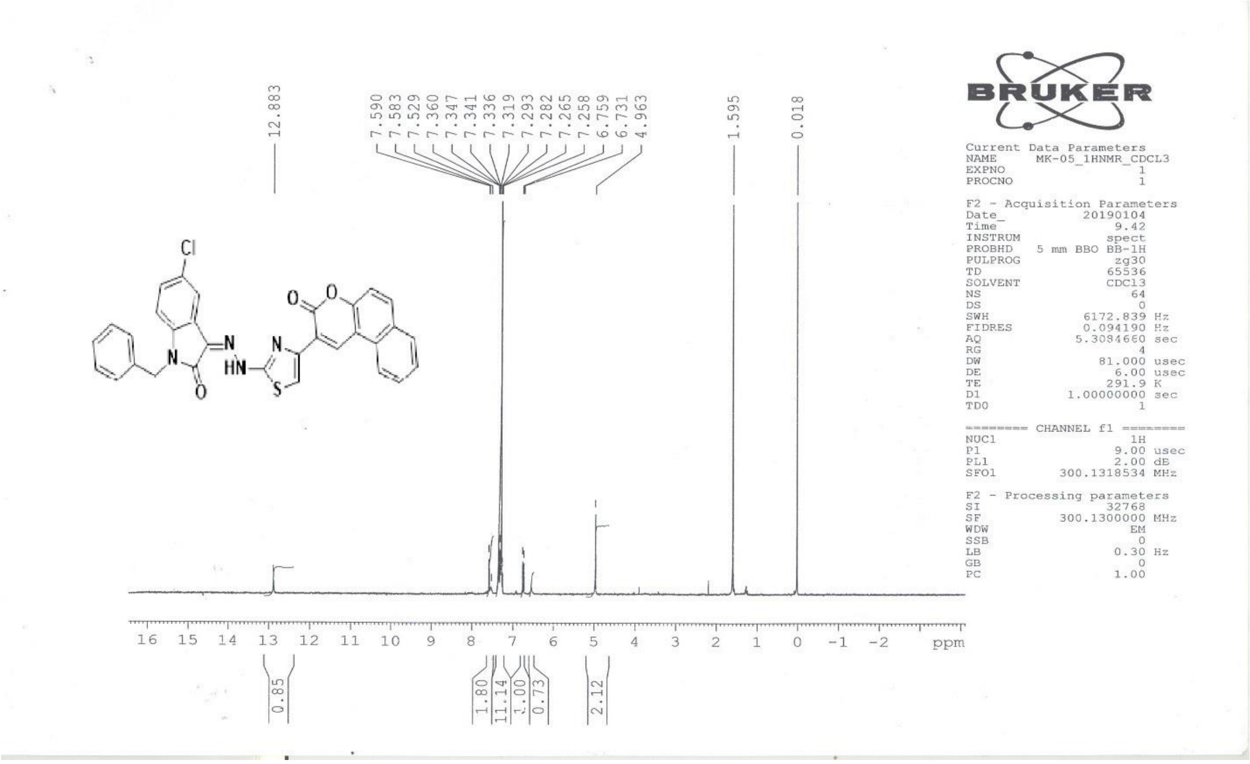

Figure S8. ^{1}H-NMR spectrum of IST-03.

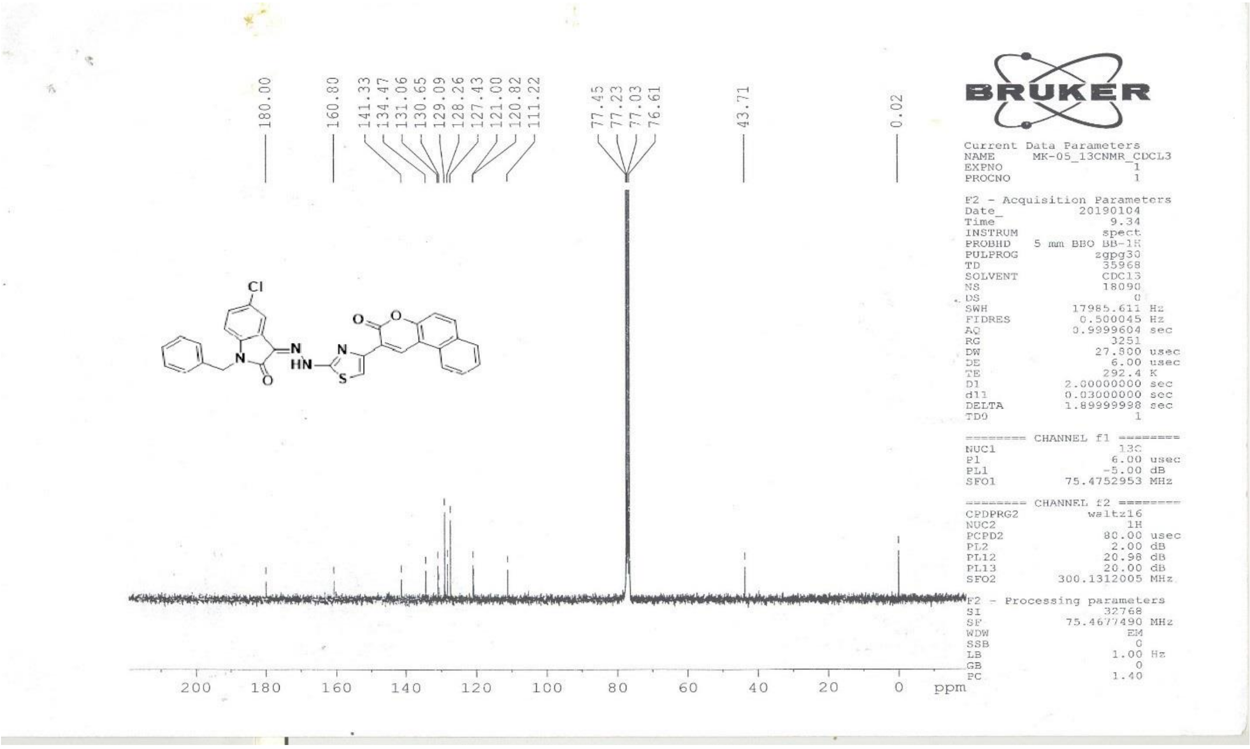

Figure S9. ^{13}C-NMR spectrum of IST-03.

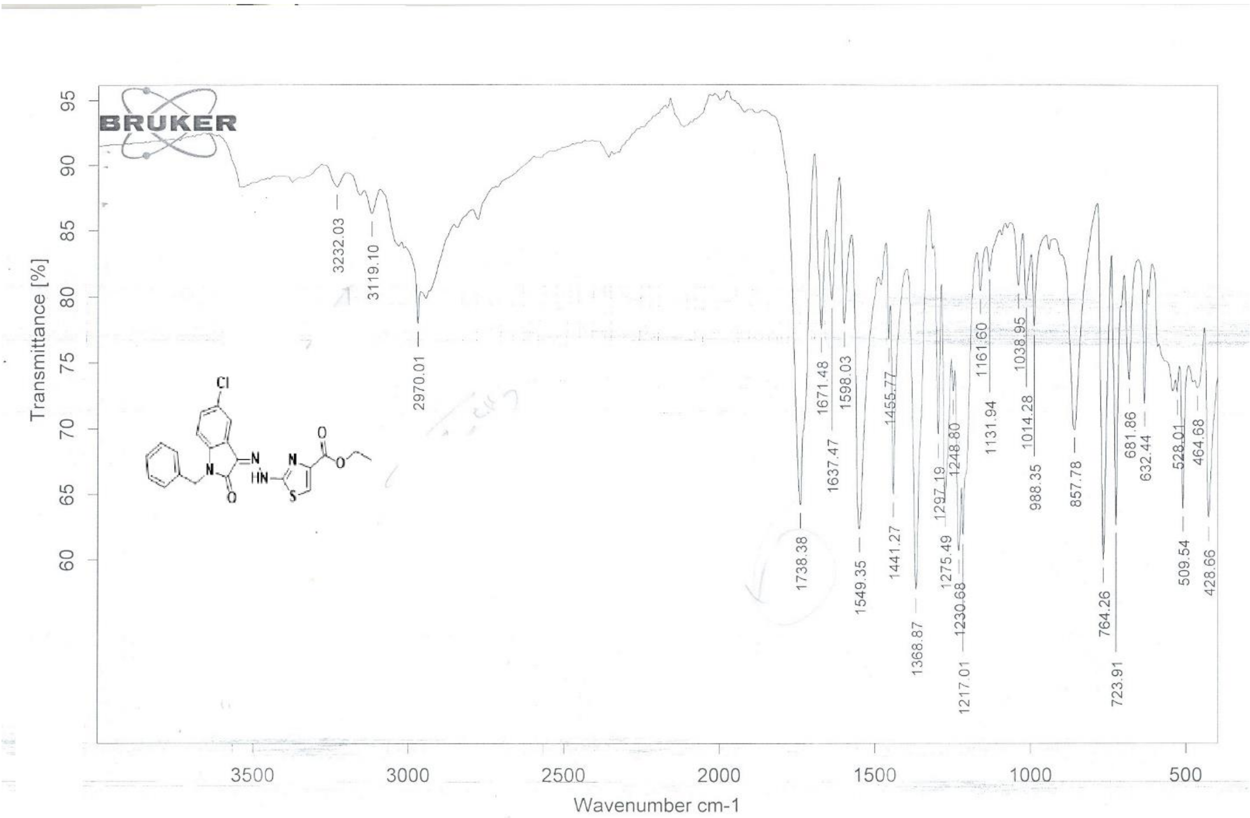

Figure S10. FT-IR spectrum of IST-04.

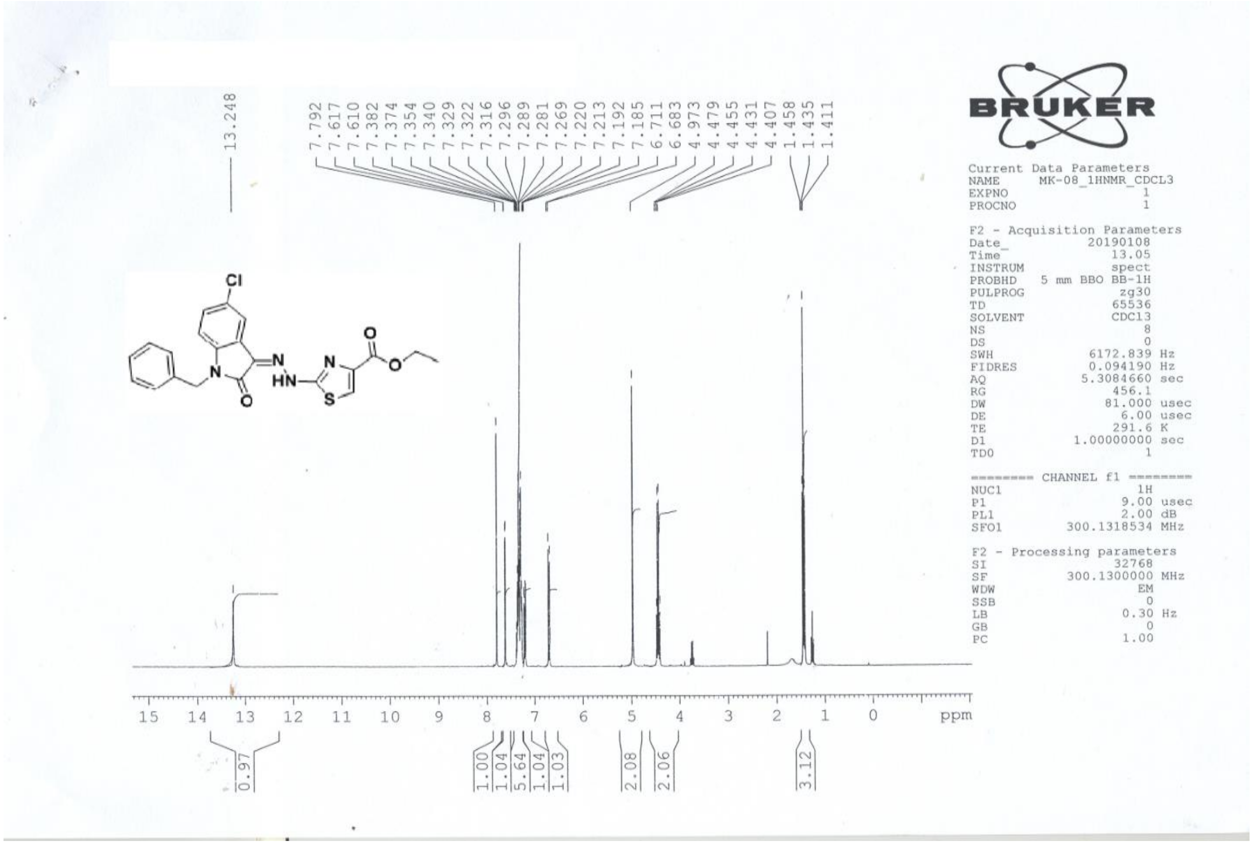

Figure S11. ^{1}H-NMR spectrum of IST-04.

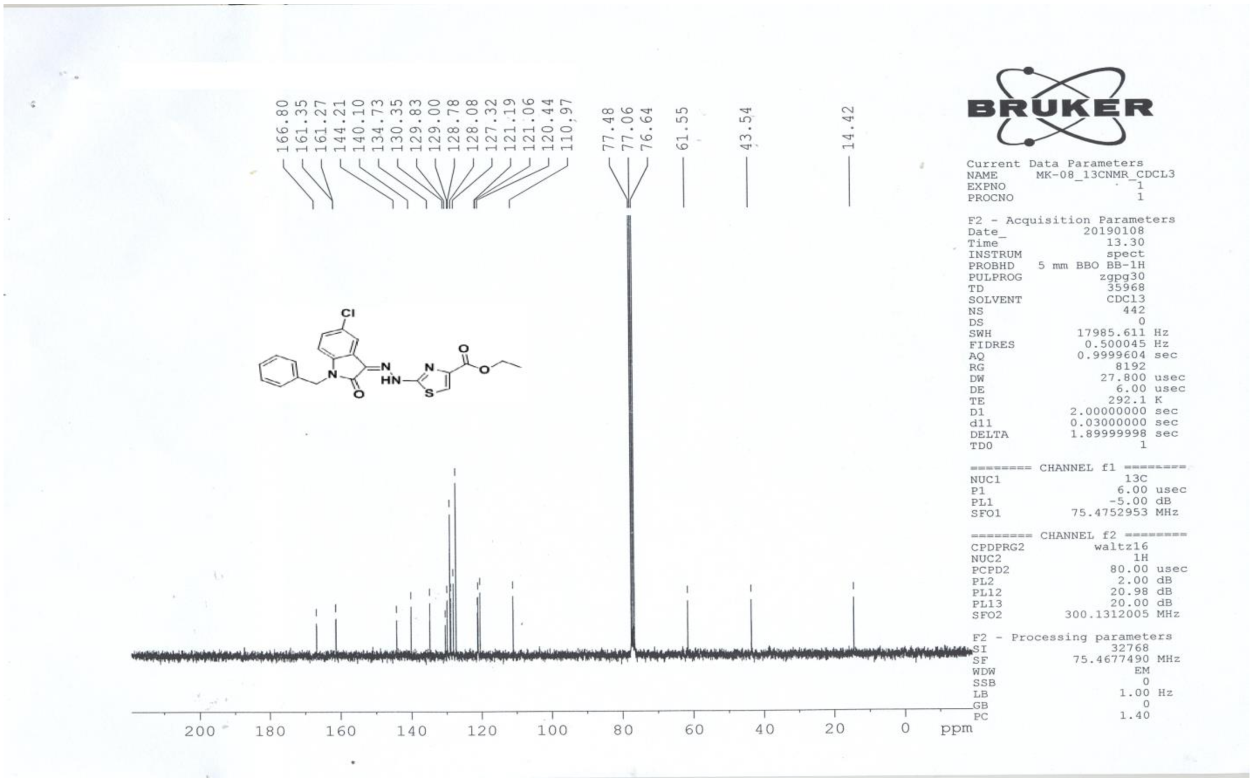

Figure S12. ^{13}C-NMR spectrum of IST-04.

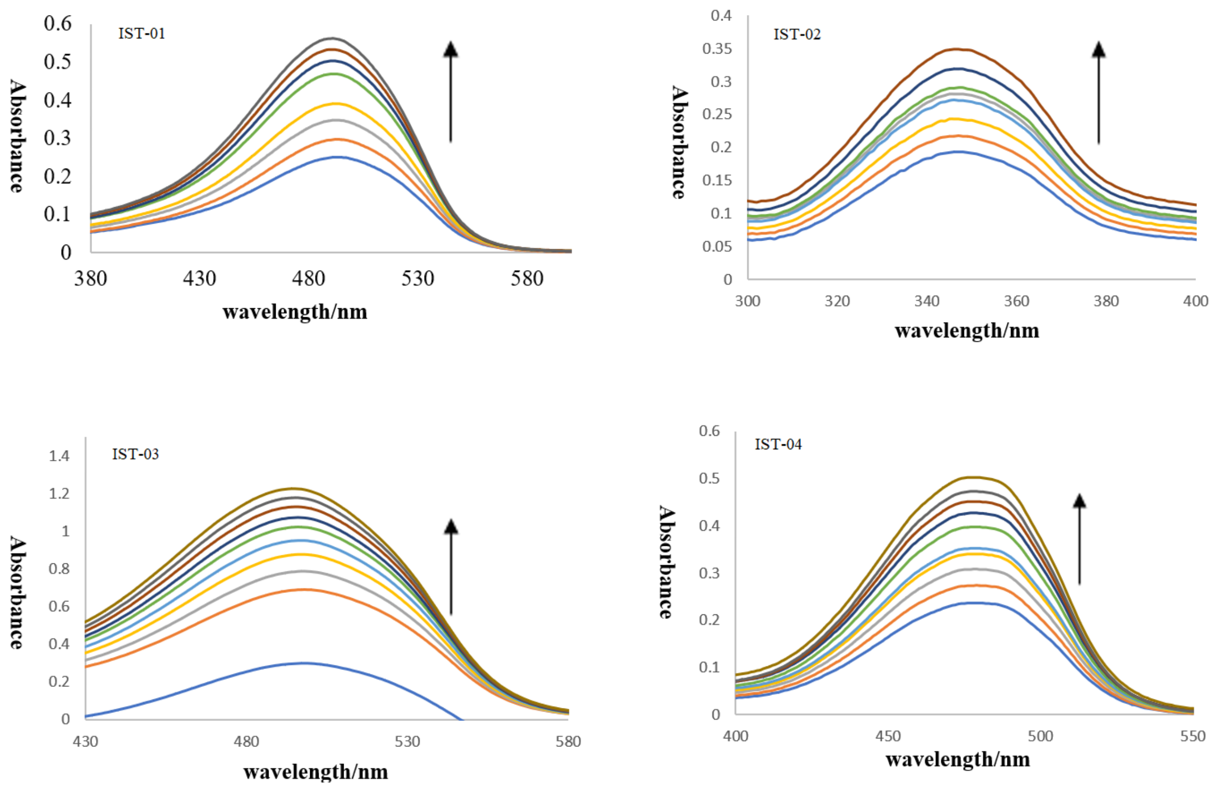

Figure S13. Concentration profiles of the synthesized compounds.

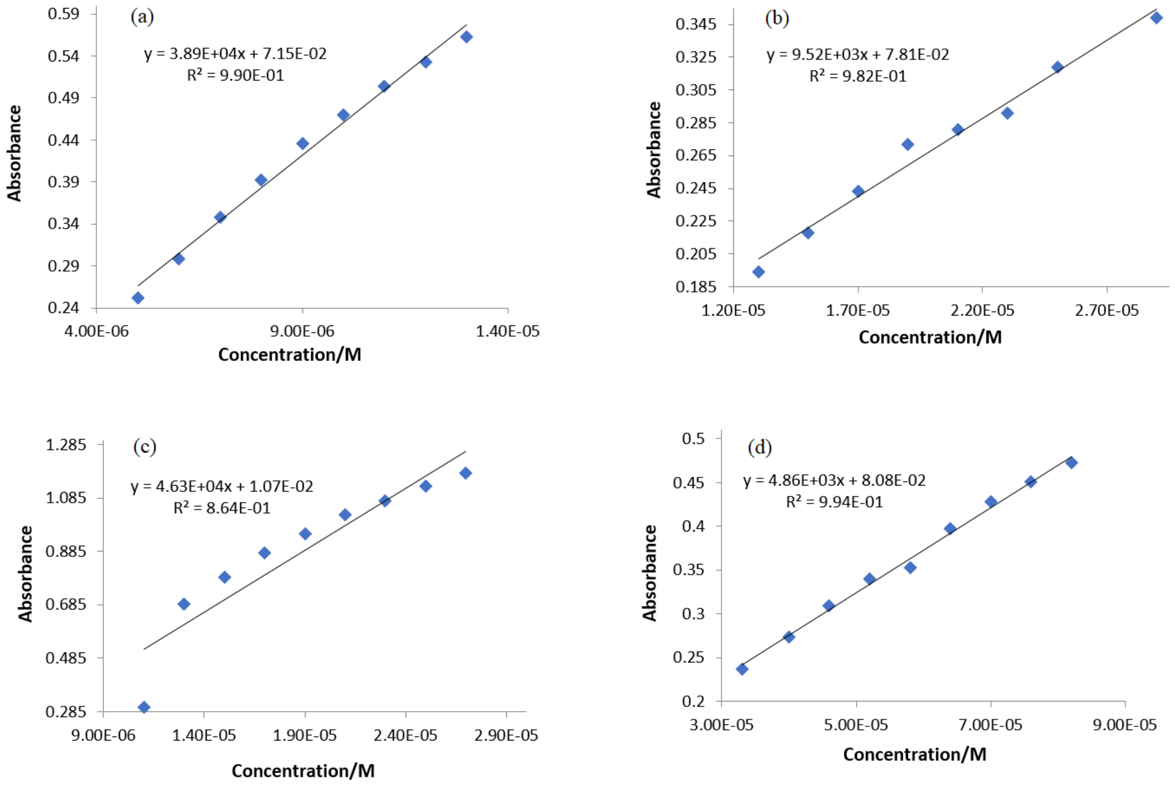

Figure S14. Absorbance vs. Concentration graphs for the determination of molar extinction co-efficient of (a) IST-01, (b) IST-02, (c) IST-03, and IST-04.

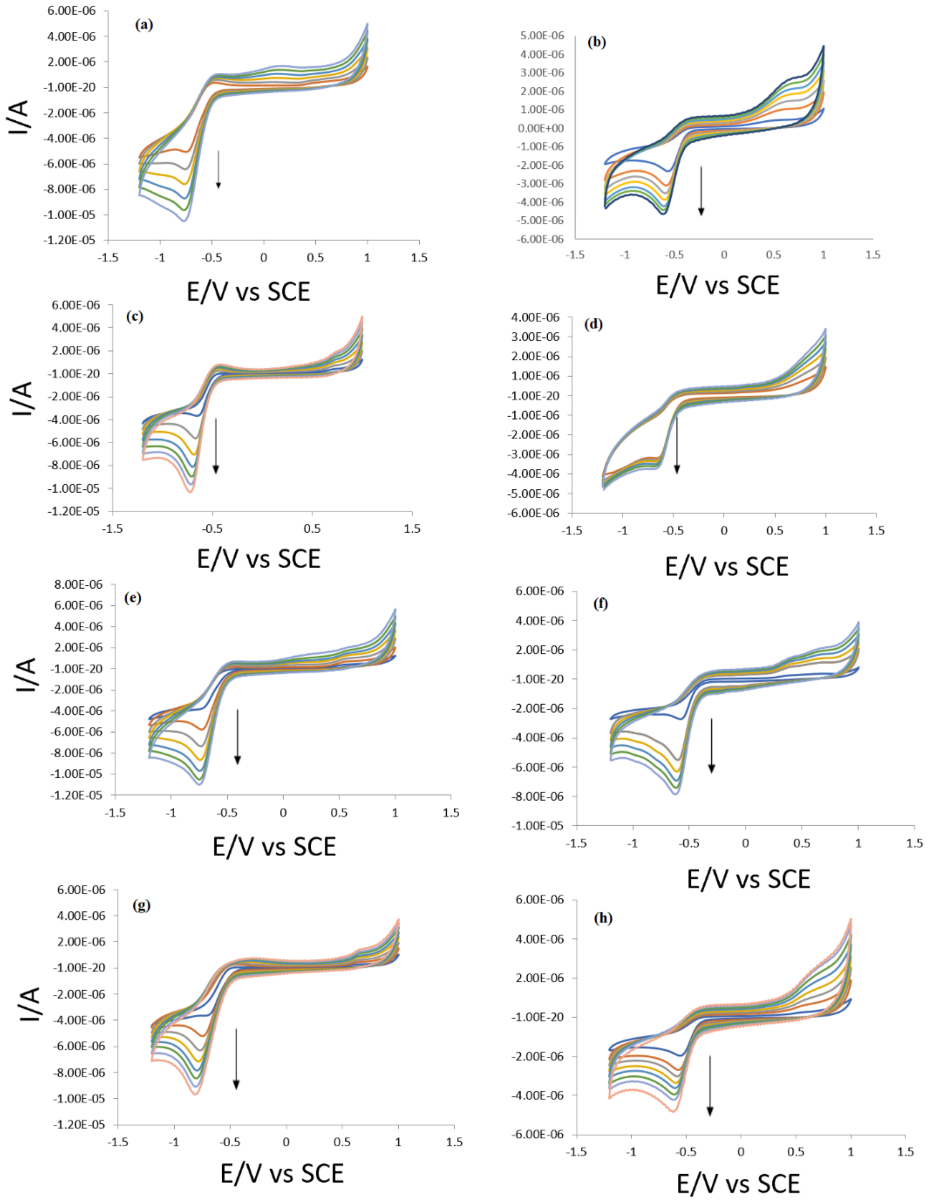

Figure S15. Overlay of cyclic voltammogram for compounds (a) IST-01, (c) IST-02, (e) IST-03, (g) IST-04 and (b,d,f,h) their DNA bound complexes at different scan rates. Arrow direction indicated increase in the scan rate as 0.03, 0.05, 0.07, 0.9, 0.11, 0.13 V/s.

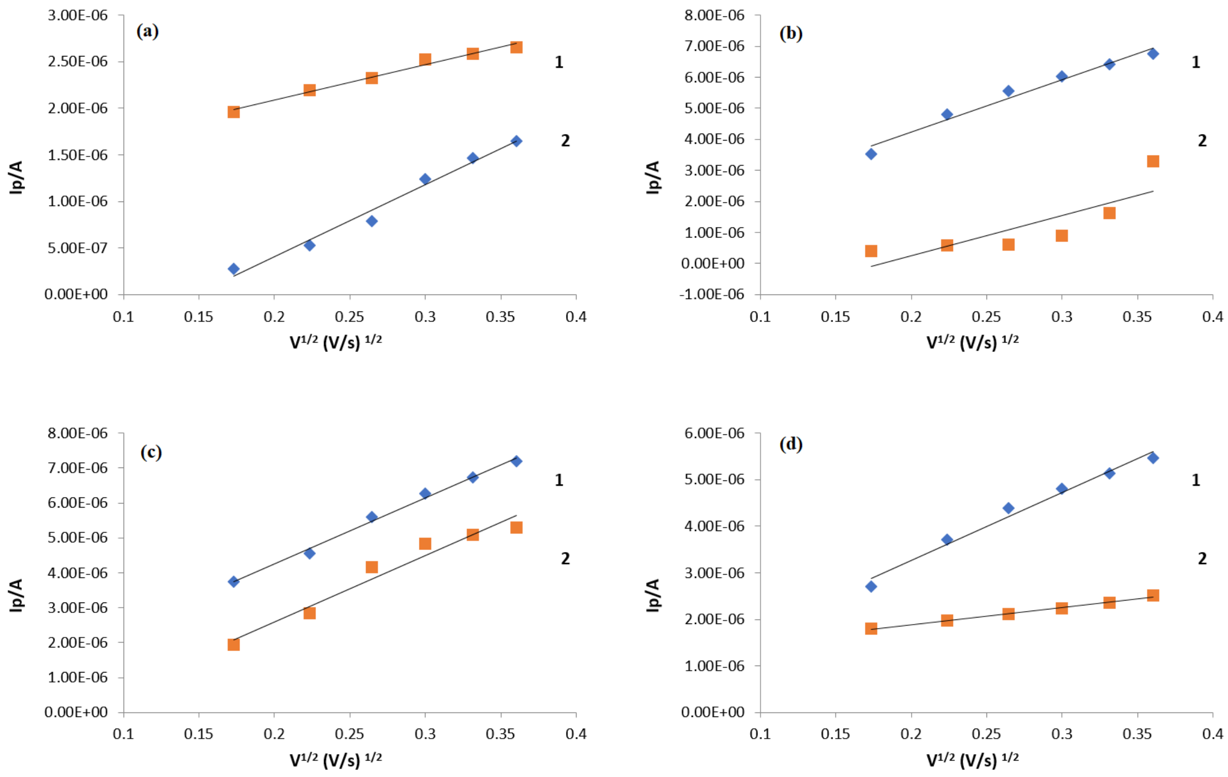

Figure S16. Plot of I_p vs $V^{1/2}$ for (a) IST-01, (b) IST-04, (c) IST-04, (d) IST-04 in the (1) absence and (2) in the presence of 60µM DNA at scan rate of 0.03, 0.05, 0.07, 0.09, 0.11, 0.13 V/s.

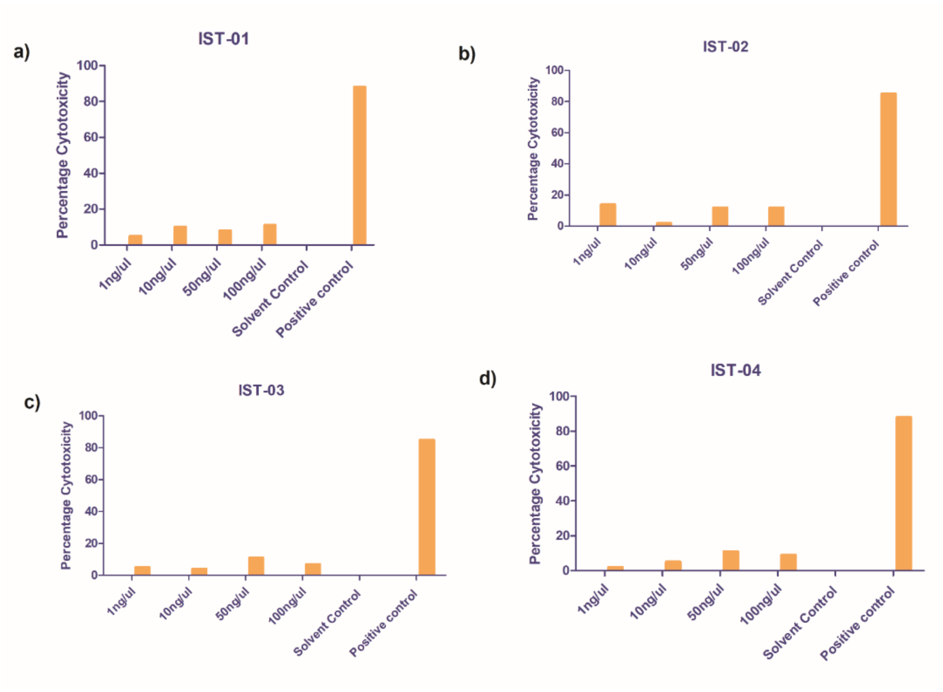

Figure S17. Percentage cytotoxicity of compounds a) IST-01, b) IST-02, c) IST-03 and d) IST-04 on HeLa cells. PBS with 10% DMSO was used as solvent control and Doxorubicin HCL was used as positive reference control.

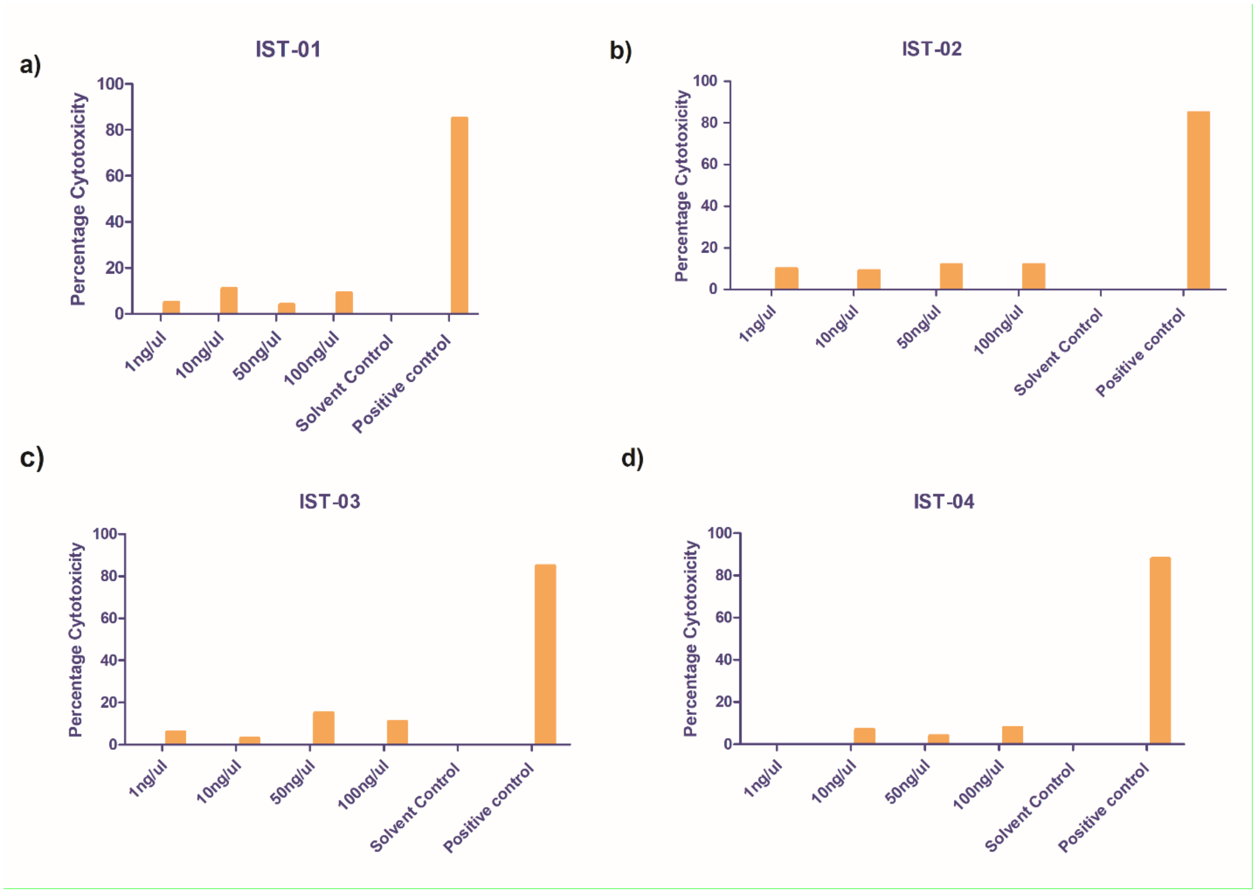

Figure S18. Percentage cytotoxicity of compounds a) IST-01, b) IST-02, c) IST-03 and d) IST-04 on MCF-07 cells. PBS with 10% DMSO was used as solvent control and Doxorubicin HCL was used as positive reference control.
